# Data-driven discovery of antiviral peptides against PRRSV using multiple machine learning models

**DOI:** 10.3389/fvets.2025.1681083

**Published:** 2025-12-05

**Authors:** Wafa Yousaf, Abdul Haseeb, Yongheng Shen, Hongquan Li, Kuohai Fan, Na Sun, Panpan Sun, Yaogui Sun, Huizhen Yang, Wei Yin, Hua Zhang, Zhenbiao Zhang, Jia Zhong, Jianzhong Wang, Nairui Huo

**Affiliations:** 1 Shanxi Key Laboratory for Modernization of TCVM, College of Veterinary Medicine, Shanxi Agricultural University, Taigu, Shanxi, China; 2 College of Resources and Environment, Shanxi Agricultural University, Taigu, Shanxi, China; 3 Laboratory Animal Center, Shanxi Agricultural University, Taigu, Shanxi, China; 4 College of Veterinary Medicine, Shanxi Agricultural University, Taigu, Shanxi, China

**Keywords:** antiviral peptides, PRRSV, mass spectrometry, machine learning, deep learning, amino acids composition, physicochemical properties

## Abstract

**Introduction:**

Cellular machinery is built upon proteins and their functional interrelationships. Their network evaluation is essential for a comprehensive insight into biological processes and may establish a foundation for predicting antivirulence. Antiviral peptides (AVPs) have robust, broad-spectrum anti-virulence capabilities. Nevertheless, the existing predicted AVPs database is insufficient and necessitates more precise, reliable annotations. This study aimed to screen differentially expressed proteins and peptides of healthy and porcine reproductive and respiratory syndrome virus (PRRSV)-infected tissues and to predict AVP’s using Machine learning and Deep learning based computational methods.

**Methods:**

Lungs, small intestine and large intestine samples were collected to validate and quantify proteins and peptides through proteomics, and followed by predicting AVPs by employing machine learning (ML) and deep learning (DL). Models were developed exploiting significant features based on physicochemical characteristics, encompassing amino acid composition (AAC), secondary structure, and hydrophilicity. Proteomics analysis facilitated peptide qualification through GO, KEGG, COG, and PPI analysis. To predict AVPs, we employed a DL graph neural network (GNN) by making its inaugural implication in this domain and benchmarked its efficacy against conventional ML random forest (RF) and support vector machine (SVM) models.

**Results:**

Findings demonstrated that lysine, arginine, and leucine were ranked nearly 0.1, highlighting their significant importance in prediction. Additionally, the correlation heatmap showed that lysine and glutamate exhibited the strongest positive association (0.57). RF model achieved an area under the curve (AUC) of 0.95 ± 2, verified via 5-fold cross-validation. In contrast, GNN and SVM models yielded 0.94 ± 1 AUC, demonstrating comparable performance across models, and revealed that the RF model outperformed compared to the others.

**Discussion:**

Integrating proteomics with computational modeling revealed peptides with antiviral potential against PRRSV. The RF model demonstrated the best discriminative power, and amino acid composition played a key predictive role. Consequently, these comparative predictive results may serve as revolutionized and distinctive resources for the experimental validation and identification of PRRSV AVPs as prospective therapeutics.

## Graphical abstract

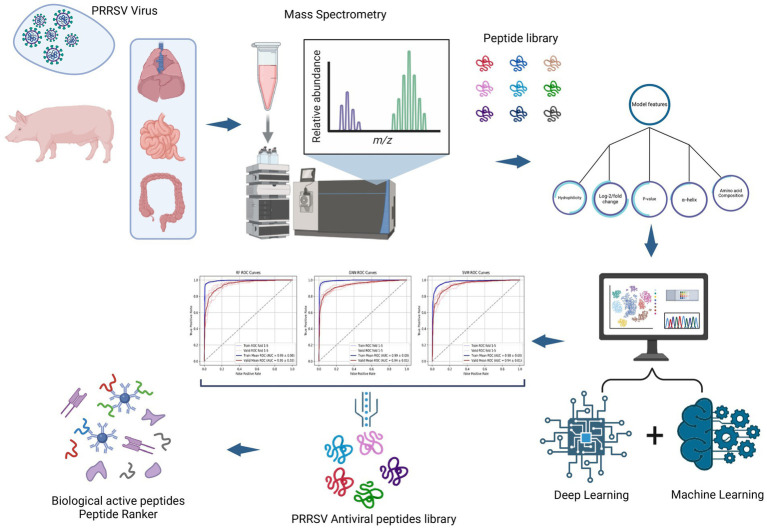

## 1 Introduction

PRRSV is an enveloped positive-sense RNA virus causing porcine reproductive and respiratory syndrome (PRRS), initially identified in Europe in 1991 and then in the United States in 1992 (1). The acute outbreaks of PRRSV are marked by significant reproductive complications in sows, perinatal fatalities, and respiratory distress in piglets (2). Viruses are formidable and widespread pathogens that induce a multitude of infectious diseases in both humans and animals (3). Genetic diversity, multiple transmission modes, and excellent host cell replication ensure persistence-assisted PRRSV and other viruses evolve (4). The porcine reproductive and respiratory syndrome virus (PRRSV) exhibits a natural capacity for environmental adaptation and evolutionary modification, resulting in considerable economic strain on the global swine sector. Due to the possibility of recombination across PRRSV genomes, the recombination between wild strains and the vaccine strains, and differences in pathogenicity, newly emerging PRRSV isolates hold significant clinical importance (5). Clinically, PRRSV infection leads to significant reproductive failure in gilts and respiratory illness in pigs of all ages, exacerbating polymicrobial disease syndromes, including porcine circovirus-associated sickness (6). The historical prevalence of viral infections has been a formidable challenge due to these traits (7). Despite the substantial development of antiviral vaccines and therapies, viral diseases persist in impacting humans and animals. Therefore, it is essential to develop a new therapeutic strategy to combat viral illnesses. So, antiviral peptides (AVPs) potentially offer a suitable treatment for PRRSV. AVPs are a subset of antimicrobial peptides (AMPs) with significant potential to protect humans and animals from numerous viral infections. Novel antivirals are needed because certain virus infections cause high morbidity and mortality, despite advances in human healthcare. Current antiviral drugs are limited by ineffectiveness, resistance, and side effects. Consequently, the burgeoning subject of “peptide-based therapeutics” targeting viruses is under investigation and appears promising (8). Antiviral peptides (AVPs) are novel therapeutic interventions for viral infections. A considerable number of decades have been dedicated to antiviral research. Antiviral agents are efficacious against numerous infections. All AVPs are derived from synthetic combinatorial databases or biological proteins and their homologs (9).

These proteins are essential in biological processes, including structural, metabolic, regulatory, and immunological functions. Modified protein function is pivotal in disease advancement, underscoring the necessity of investigating proteome anomalies within the framework of pathology (10). Numerous independent proteomics and mechanistic investigations of PRRSV infection indicate the dysregulation of innate immune signaling, autophagy/lysosomal pathways, the ubiquitin-proteasome system, NF-κB/TLR, and apoptosis-related pathways; these pathways are recognized as pivotal to antiviral responses and are often influenced by antiviral/antimicrobial peptides (AVPs/AMPs). PRRSV modifies TBK1/IFN-I (11) signaling through autophagy and significantly disrupts UPS and autophagy-related mechanisms (12–14). In contrast, antiviral peptides like LL-37 and other (AMPs) exert effects directly on viruses through membrane and entrance inhibition, as well as indirectly by altering type-I interferon signaling, ISG/ISG15 pathways, autophagy, and NF-κB/TLR signaling.

As the transcriptome data, encompassing mRNA levels, are insufficient for deducing protein abundance; hence, direct assessments of protein activity are often necessary (15). Conventional approaches for selecting therapeutic proteins typically concentrate on a limited number of targets. Recent advancements in mass spectrometry have facilitated extensive proteome-wide studies. The expansion of proteomics has driven progress in bioinformatics, linking protein regulation, phenotypic expression, and the initiation and progression of diseases (16).

Mass spectrometry-based bottom-up proteomics facilitates the analysis of complicated proteomes. Recent technological advancements, including GO (17), PCA, PPI, and KEGG, have enhanced proteome depth and throughput (18) up to cellular components and biological processes influencing pathways (19). The domain has undergone substantial technological improvement, exemplified by new, robust, high-capacity liquid chromatography (LC) systems and cutting-edge mass spectrometers that enable peptide separation by ion mobility (20). Moreover, these developments coincided with the evolution of high-throughput data collection procedures and a progression in computing techniques for proteomics data analysis (21). Enabled by advancements in computational hardware and programming frameworks, computational proteomics has developed into a distinct, multidisciplinary domain (22). Effective data visualization is essential for interpreting data and conveying the outcomes of increasingly intricate research (23). Numerous data analysis tools incorporate visualization capabilities to address this need; yet, visualization often does not serve as a key emphasis in the development of innovative analytical data workflows and is generally considered a secondary priority (24). Consequently, data assessment, interpretation, and visualization often reside within the purview of experts who possess a comprehensive grasp of the data and are adept in its computational exploitation (25). Although numerous reviews focused on individual software tools or analyzed the technical aspects of the visualization process by summarizing existing R libraries, but they did not effectively elucidate proteomics, the significance of specific visualizations, and methods for their interpretation (26).

Regarding the proteomics domain, the identification of AVPs offers an effective approach for treating virus-infected cells. The recent advancement of peptide-based therapeutic medicines through machine learning and deep learning techniques has emerged as a significant focus of research owing to its encouraging outcomes (27). These techniques are essential in the advancement and development of antiviral peptides and peptidomimetics, notably through the creation of specialized databases like DRAVP, AVPdb, and DBAASP. These resources enable AVP characterization but encounter limitations and challenges, including small datasets, incomplete annotations, insufficient integration with multi-omics data, issues such as overfitting, restricted experimental validation, and a deficiency in mechanistic insights that impede clinical translation (28). Recent advancements in machine learning (ML) and deep learning (DL) methodologies have markedly improved the prediction and design of antiviral peptides (AVPs). These computational methods provide the detection of nuanced sequence patterns, physicochemical characteristics, and structural attributes linked to antiviral efficacy, providing superior accuracy and efficiency relative to traditional experimental screening. Numerous recognized machine learning and deep learning based (SOTA) state of the art tools, such as GAN (29), AVPpred (30), ClassAMP (31), iAMP-2 L (32), Meta-iAVP (33), AntiVPP 1.0 (4), iAMP-CA2L (34), AI4AVP (35), DGM (36), and Deep-AVPpred (37), have proven the efficacy of these methods in expediting the finding and characterization of peptides, specifically AVPs and AMPs. Based on these tools, the current study utilizes the latest analogous computational methods to improve the identification and prediction of prospective antiviral peptides (38). This study aimed to examine and predict antiviral peptides, which possess significant potential in drug discovery but have made minimal advancement in prediction. The physicochemical properties of peptides facilitate the identification of antiviral peptides. A prior study indicated that sequence-derived physicochemical properties could forecast antimicrobial peptides (AMPs) (39). Key physicochemical properties may encompass amino acid composition, the secondary structure of the peptide, and hydrophilicity. These characteristics can provide a foundational framework for the creation of machine learning or deep learning algorithms designed to predict antiviral peptides (30). A variety of Python-based machine learning algorithms have been employed in scientific research to forecast antiviral peptides. SVM (support vector machine) is one of the most recently used models based on the physicochemical properties of amino acids (40). The support vector machine (SVM) algorithm is also among the most prevalent machine learning (ML) techniques for forecasting active substances and chemical characteristics (41). The precision of SVM compound classification and its non-linear regression capabilities for virtual screening render it a significant tool in cheminformatics machine learning. Support Vector Machines (SVM) can classify data, identify outliers, and do regression analysis through structural risk minimization. Its utility in pharmacological research is unparalleled, facilitating virtual screening, drug-target interaction prediction, and the identification of novel targets (42). Meanwhile, some recent studies have also employed the RF model to differentiate antiviral and non-antiviral properties of peptides based on physicochemical properties like amino acid composition AAC, primary and secondary structure, etc. The random forest (RF) model is the most widely used machine learning approach for antiviral peptides (AVP’s) prediction (43). The random forest algorithm serves as a supervised learning system. The name indicates, “This is a method of generating a forest from multiple viewpoints to achieve randomness.” The principal advantage of the random forest technique includes its applicability to both regression and classification problems (44).

Along with machine learning models, modern deep learning algorithms can also be employed to forecast antiviral drugs. Top-notch identification tool, deep learning graph neural network (GNN) model promotes antimicrobial peptide drug discovery and design using structural and sequential AMP information (45). It significantly helps to classify graphs and nodes (46) and successfully conveys molecular structures and traits. Stacking convolution and attention operations and applying sigmoid or softmax functions for classification represent network structure features and adjacency matrices (47). Unlike sequence-based or array-based neural networks, these models use features and node connections to increase information extraction and accuracy (48). This research represents the inaugural use of a deep learning-based Graph Neural Network (GNN) model for predicting antiviral peptides (AVPs) derived from proteomics data. Although conventional machine learning methods like Random Forest (RF) and Support Vector Machine (SVM) have been utilized for AVP classification tasks, a comparative analysis of Graph Neural Networks (GNN) against these existing models remains unexamined. By integrating GNN with RF and SVM, we provide a thorough performance evaluation, underscoring the capability of GNNs to discern intricate structural and relational characteristics of peptides that traditional models may neglect.

## 2 Materials and methods

### 2.1 Animals and sampling

The animal study was approved by the Laboratory Animals Ethics Committee of Shanxi Agricultural University (Approval number: SXAU-EAW-2023P.FU.004007363). A total of six female 30-day crossbred piglets were raised in the experimental management center of Shanxi Agricultural Center, with an excess of water and feed as per internationally recognized standards. Three piglets, with each animal receiving 2 mL of nasal drops inoculum containing 10^6^ TCID₅₀/mL of the PRRSV virus on the 60^th^ day. The remaining three piglets were considered the control group. At the 81^st^ day, tissue samples were collected from lungs, large intestine, and small intestine of all the healthy and PRRSV infected pigs, subsequently categorized into six distinct groups: KD (large intestine of healthy control pig), BD (large intestine of virus-infected pig), KX (small intestine of healthy control pig), BX (small intestine of virus-infected pig), KF (lungs of healthy control pig), and BF (lungs of virus-infected pig). Three samples were concurrently taken for each organ group. Protein and peptide identification and quantification were conducted using mass spectrometry by tgene Biotech, Shanghai, China.

### 2.2 Sample preparation

50 mg of sample from each tissue was collected and crushed, the correct volume of lysis solution (8 M urea/100 mM Tris-Cl, pH 8.0) was added. The mixture was centrifuged at 12000 × g for 5 min. The clarified supernatant was filtered using ultrafiltration tubes with a 10KD pore size. The ultrafiltered solution was collected, and the pH of the solution was adjusted to 6.0. The supernatant was then centrifuged again at 12000 × g for 5 min to remove salt. After removing salt from the supernatant, the peptide solution was centrifugally concentrated, dried, and kept at −20 °C for subsequent mass spectrometry analysis.

### 2.3 Mass spectrometry

Mass spectrometry was employed to detect samples on a thermal ultimate 3,000 RSLCnano nanoliter liquid tandem Q Exactive HF spectrometer. Peptide samples were fed into an autosampler, bound to a C18 trap column (75 μm*2 cm, 3 μm particle size, 100 Å pore size, Thermo), and then separated on a handmade analytical column (75 μm*25 cm, 1.9 μm particle size, 100 Å pore size). A mobile phase A (0.1 percent formic acid, 3% DSO, and 97% water) was used to create an analytical gradient. The flow rate of analysis was set at 300 nL/min, and mass spectrometry was carried out in DDA mode. The MS1 full scan parameters were set to resolution 60 K@200 m/z, scanning range 350–1,500 m/z, and a maximum injection time of 30 ms. The MS2 scan parameters were set as follows: resolution 15 K@200 m/z, AGC target 1E5, and maximal injection time 50 ms. The maximal injection time was set at 30 s.

### 2.4 Feature representations for RF, SVM, and GNN model

Choosing the right features is essential for creating precise predictive models since the effectiveness of the model relies on the selected features. Identifying antiviral peptides involves the careful selection of essential attributes to validate candidates derived from mass spectrometry data. Five characteristics were chosen to determine the peptides with the greatest potential for antiviral activity. Prior to validation, the peptides dataset using the machine learning RF, SVM, and deep learning GNN models, the dataset was refined based on the *p* value, i.e., *p* < 0.05, and Log2/fold change value, which we set to a 3-fold change. *p*-value represents the statistical significance of differential expression between groups, with a threshold of p < 0.05 considered significant. The log₂(fold change) denotes the magnitude and direction of expression differences, where positive values indicate upregulation and negative values indicate downregulation. The other features were based on physicochemical properties and were formulated for the RF, SVM, and GNN models encompassing AAC (Amino acids composition), Secondary structure, Polar charge, and hydrophilicity. These five distinctive characteristics mentioned were the backbone for the prediction of antiviral peptides by employing a machine-learning RF and SVM model and a deep learning GNN model.

### 2.5 Datasets

The antiviral peptide and non-antiviral peptide datasets used in this study were sourced from *Thakur et al.*’s study database for the model calibration, which validated 1,056 peptides experimentally, comprising 604 highly effective antiviral peptides and 452 ineffective ones. Each peptide segment in this dataset has a distinct feature sequence identifier. To address the common issue of class imbalance in machine learning model training, we employed a mixed sampling approach on this dataset, generating a new data collection totaling 1,094 samples to achieve a balanced distribution of positive and negative samples.

### 2.6 Model architecture and predictive framework

The sequence data of antiviral and non-antiviral peptides were selected from our proteomics database following the filtration of peptide data based on fold change and *p*-value. The peptide chains were split, and the frequency of single-letter codes was enumerated. This data, combined with labels, created a training set consisting of 1,094 samples, which were divided into 80% training sets and 20% validation sets, respectively, according to the 5-fold cross-validation rule. These data sets were fed into random forest (RF), support vector machine (SVM), and graph neural network (GNN) models for training and validation. Initially, machine learning models demonstrated emerging correlations between the frequency distributions of single-letter codes and their corresponding sample labels. As the investigation advanced, complex patterns and interdependencies among these codes were revealed, providing greater insights into their roles in influencing model predictions. Finally, the accuracy of each model was evaluated individually and compared, and the model exhibiting the highest overall accuracy was selected to predict labels for the unlabeled dataset. Table 1 illustrates the association between single-letter codes and amino acid nomenclature.

**Table 1:** Amino acids representation by single letter and three letters for the construction of RF, SVM, and GNN models based on amino acids composition feature.

One-letter	Three-letter	Amino acid name	One-letter	Three-letter	Amino acid name
A	Ala	Alanine	N	Asn	Asparagine
B	Asx	Aspartic/Asparagine	P	Pro	Proline
C	Cys	Cysteine	Q	Gln	Glutamine
D	Asp	Aspartic acid	R	Arg	Arginine
E	Glu	Glutamic acid	S	Ser	Serine
F	Phe	Phenylalanine	T	Thr	Threonine
G	Gly	Glycine	U	Sec	Selenocysteine
H	His	Histidine	V	Val	Valine
I	Ile	Isoleucine	W	Trp	Tryptophan
K	Lys	Lysine	X	Xxx	Any
L	Leu	Leucine	Y	Tyr	Tyrosine
M	Met	Methionine	Z	Glx	Glutamine/Glutamic

### 2.7 Model introduction (partial research methods)

#### 2.7.1 Random forest

Random forest (RF) is a machine-learning algorithm based on an ensemble of decision trees. It improves the classification accuracy of the model by constructing multiple decision trees and aggregating their prediction results. This method employs bootstrap sampling to generate diverse training subsets and randomly selects feature subsets during node splitting. This dual randomness mechanism makes it particularly well-suited for handling high-dimensional biological data. In this study, we optimized the parameters through grid search and 5-fold cross-validation, ultimately determining the optimal parameter combination as follows: n_estimators = 180 (number of decision trees), max_depth = 10 (maximum depth), min_samples_split = 5 (minimum number of samples required to split an internal node), and min_samples_leaf = 1 (minimum number of samples required to be at a leaf node). This parameter setting effectively captures the complex nonlinear relationships between features and labels while avoiding overfitting by constraining the depth of the trees and the conditions for splitting.

#### 2.7.2 Support vector machine

Support vector machine (SVM), as a robust supervised learning model, successfully achieved effective distinction between viral peptides and antiviral peptides in this study by constructing an optimal classification hyperplane in a high-dimensional feature space. The core advantage of this algorithm lies in its ability to handle complex nonlinear classification problems through the kernel trick. Through systematic grid search and cross-validation, the optimal parameter combination was ultimately determined as follows: kernel = ‘rbf’, C = 2, and degree = 6. This parameter setting not only ensures the model’s capability to express nonlinear relationships but also appropriately controls model complexity via regularization strength. The final model demonstrated stable classification performance on the test set, providing a reliable classification benchmark for subsequent functional studies of viral peptides.

#### 2.7.3 Graph neural network

Graph neural network (GNN) is a deep learning model suitable for graph-structured data. Graph neural networks have gained significant interest in biology due to their ability to represent complex interactions in molecular and protein structures. By modeling amino acid sequences as graph structures, GNN effectively elucidates the patterns of structure and interaction, thus aiding in the identification of potential interactions between viral peptides and antiviral peptides. GNN can learn rich representations of high-dimensional features by sending messages and updating the features of nodes (amino acids) and edges (relationships between amino acids), thereby enhancing the model’s expressive power. Training on data sets with GNN not only reveals key amino acid features associated with antiviral activity but also provides new perspectives for understanding the complex relationships between biomolecules.

**Figure 1:**
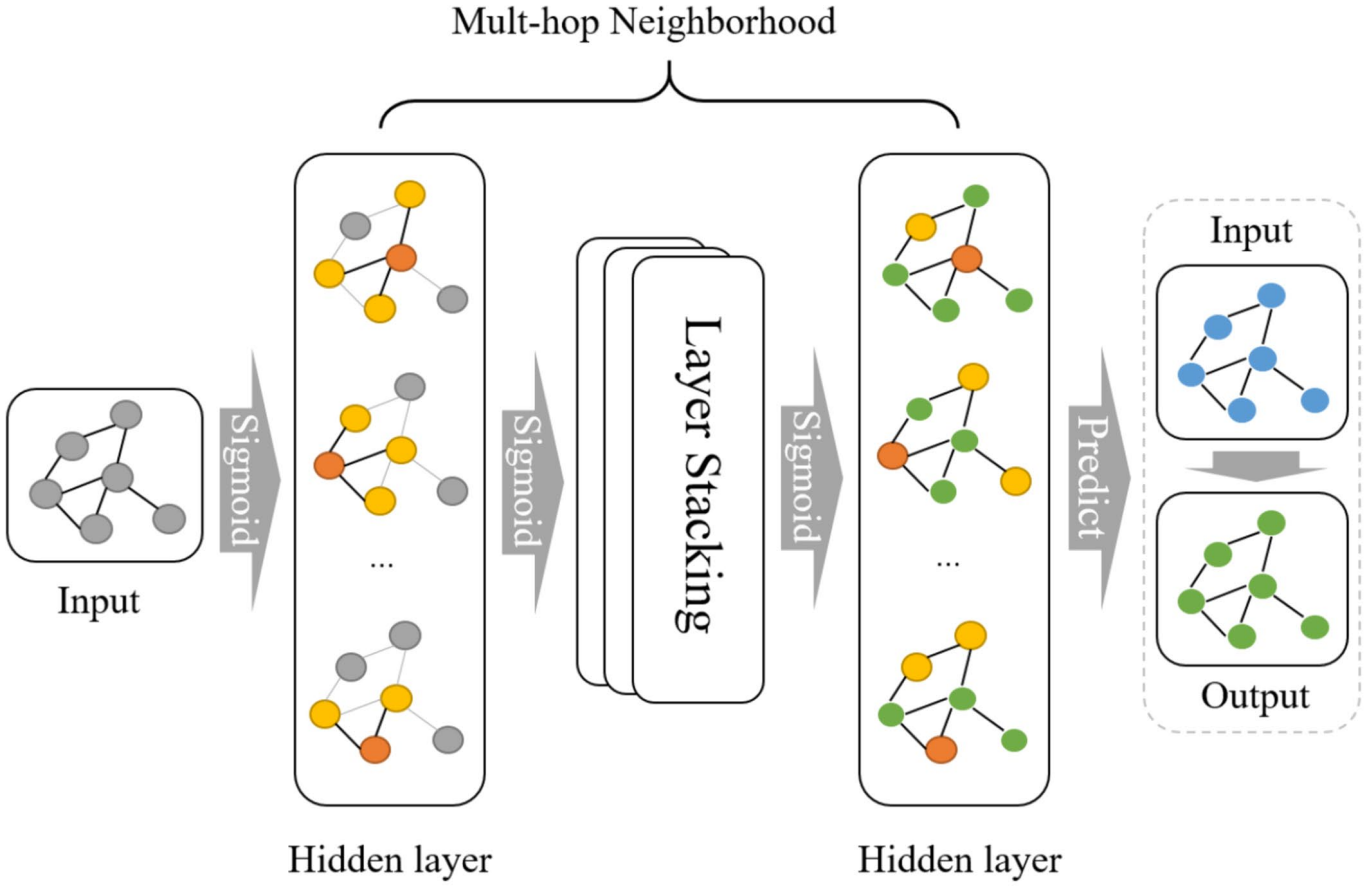
Operation principle of the GNN model.

In this study, during training set construction, we defined each amino acid sequence as a node, with each node retaining information about the composition of various amino acids. Labels were denoted as 0 or 1 to indicate classifications of viral peptides and antiviral peptides, establishing graph-structured data. The prediction set maintains the same structure as the training set but lacks labels for prediction purposes. During model operation, the initial layer applies an activation function to aggregate information between nodes in the graph structure in the form of edges. After several layers of aggregation and multiple rounds of multi-hop neighborhood operations, the model expands its receptive field to acquire global information and ultimately retains the weight matrix. Subsequently, the prediction set with the same graph structure is loaded into the model, applying the trained weight matrix to derive the final prediction results Figure 1.

#### 2.7.4 GNN construction layout

The application of GNN in this study began with the construction of graph-structured data. Initially, the 24-dimensional amino acid frequency features of peptide sequences were partitioned into four functional groups based on their physicochemical properties (AAC, hydrophilic, secondary structure, and Polar charge) (S2). Fully connected edges were established between these groups to represent inter-group interactions, resulting in graph-structured data comprising four nodes, each with six-dimensional features.

**Figure 2:**
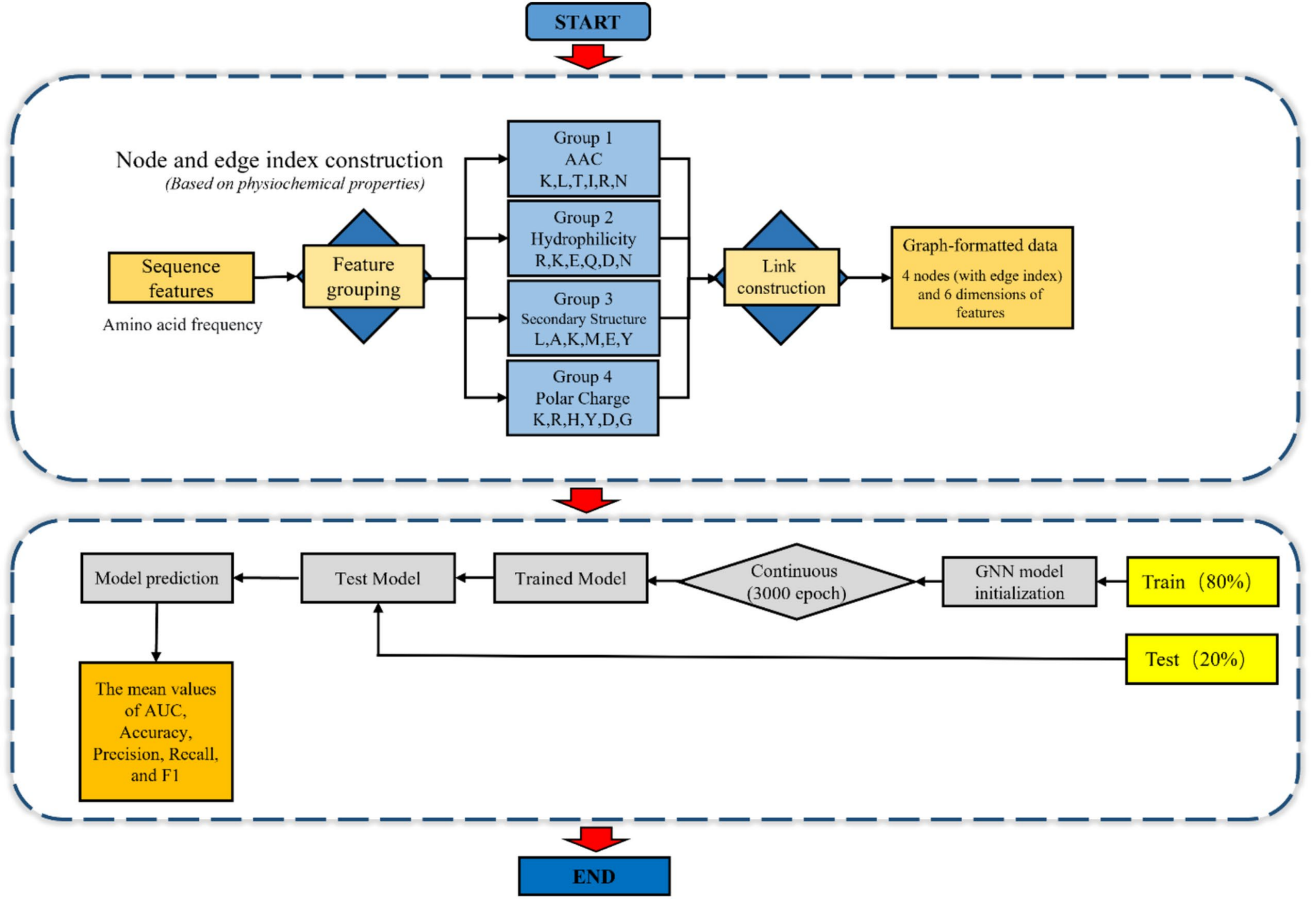
Step by step figure of node formation and amino acids features data input of the schematic layout for the GNN model in AVPs prediction.

Subsequently, the GNN model was initialized and trained over 3,000 iterations. A 5-fold cross-validation approach was employed for model optimization and evaluation, where each fold utilized 80% of the data for training and 20% for validation. Model performance was comprehensively assessed using the mean values of AUC, accuracy, precision, recall, and F1-score derived from the cross-validation results (Figure 2).Pseudocode for AVP peptides classification using GNN:
*Start*1: Load peptide dataset from CSV file
2: Define amino acid feature grouping based on biochemical properties
3: Rearrange features according to predefined groups Graph Construction
4: *FOR* each peptide sample in the dataset
5: Create graph nodes from feature groups (4 nodes, 6 features each)
6: Build linear chain edges between adjacent nodes
7: *END FOR*Model Setup
8: Initialize GNN model with 2 graph convolution layers
9: Set training parameters: 3000 epochs, learning rate 0.001 Cross-Validation Training
10: *FOR* each fold in 5-fold cross-validation
11: Split graph data into training set (80%) and validation set (20%)
12: *FOR* epoch = 1 to 3,000
13: Train GNN model on training graphs
14: Update model parameters via backpropagation
15: *END FOR*
16: Evaluate model performance on validation set
17: *END FOR* Output
18: Calculate average performance metrics across all folds
19: Generate ROC curves for model evaluation
*End*


### 2.8 Schematic layout

**Figure 3:**
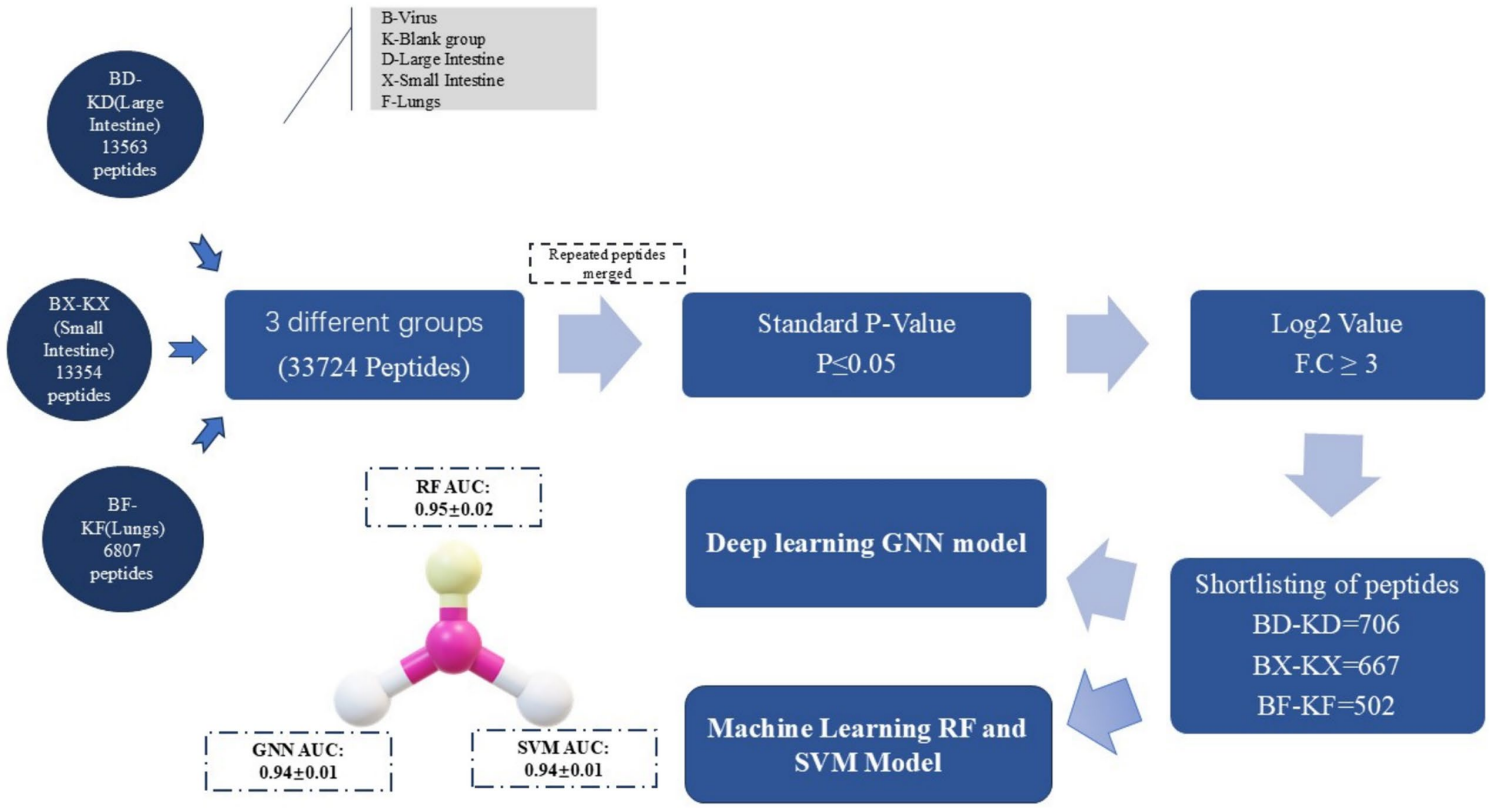
Schematic layout of AVPs prediction: rough peptides data collection via mass spectrometry and refining it based on the *p*-value, log2 value, DL & ML models trained with significant features.

The following figure represents the schematic view of the PRRSV antiviral peptides prediction with the aid of machine learning RF and SVM models and the deep learning GNN model (Figure 3).

## 3 Data analysis

The mass spectrometry data were processed by MaxQuant (V1.6.6), which used Andromeda’s database search approach and the *sus scrofa* proteome UniProt database. The GO analysis, KEGG route, and COG of the proteins and peptides were generated using the eggnog-mapper software’s Diamond program. Later, the peptides were shortlisted based on their ideal *p*-value and fold change/Log-2 value, and antiviral peptides were predicted using RF, SVM, and GNN models. The comparative accuracy of all three models was validated based on each model’s training and validation receiver-operating characteristic (ROC) curve individually.

## 4 Results

### 4.1 Quantitative analysis of peptides

**Figure 4:**
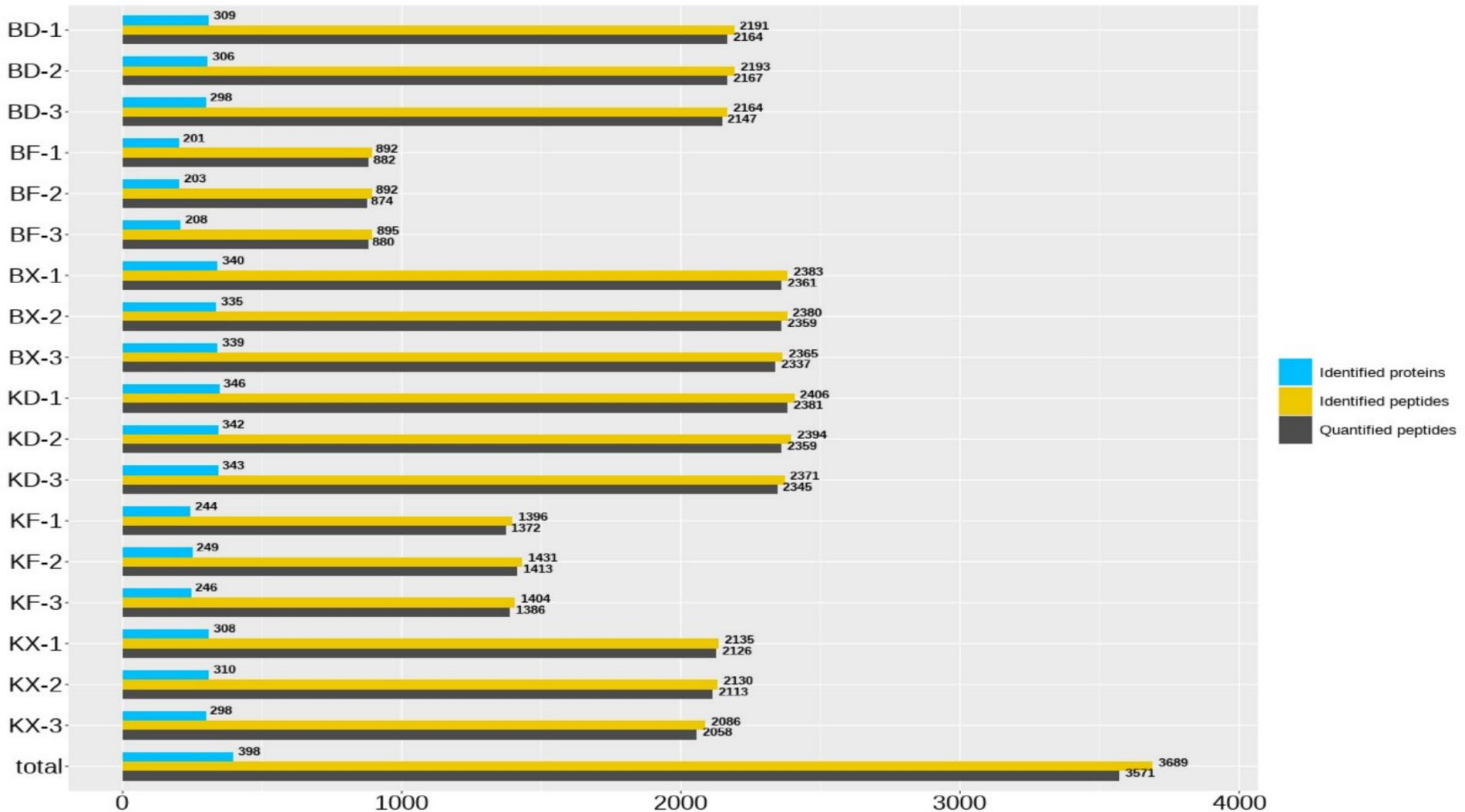
Proteins and peptides quantification analysis of the collected samples.

The selected samples were collected, processed, and analyzed using mass spectrometry to quantify the proteins and peptides. Because different proteins and peptides examined from different groups could have the same sequence, the peptides and proteins with the same sequence were merged. Table 2 and Figure 4 demonstrate the quantitative information of proteins and peptides derived from mass spectrometry analysis of samples from all groups.

**Table 2:** Mass pectrometry-based quantified proteins and peptides numerical data.

No	Group	Quantified proteins	Quantified peptides
1	BD	913	6,478
2	BF	612	2,636
3	BX	1,014	7,057
4	KD	1,031	7,085
5	KF	739	4,171
6	KX	913	6,297

## 5 Differential peptides screening

**Figure 5:**
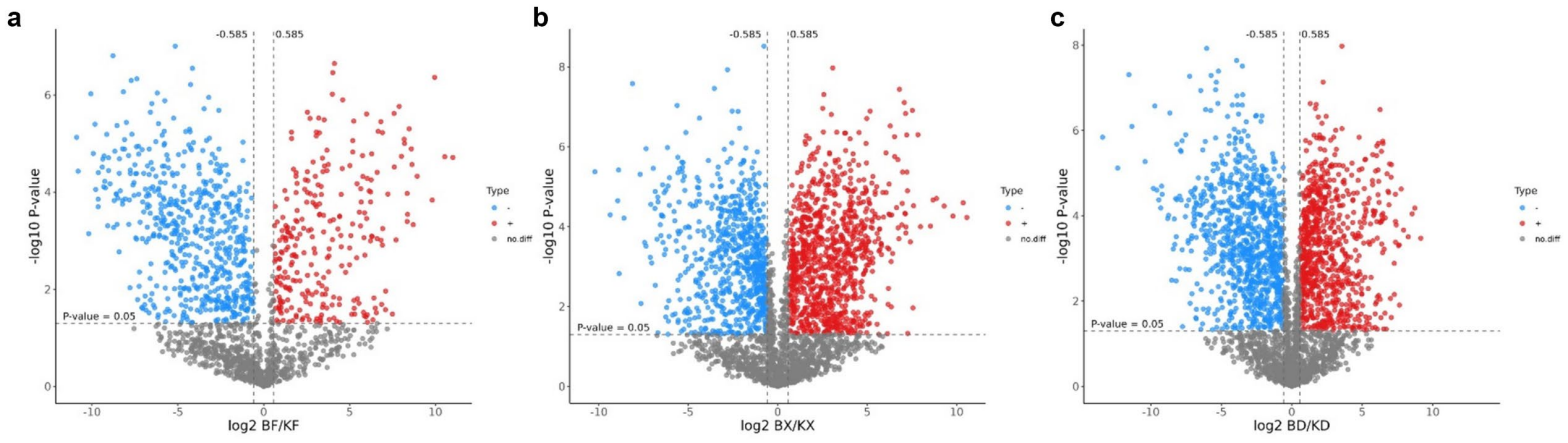
Volcano plot of differential peptides of all groups. Red color indicates up-regulated peptides, blue color indicates down-regulated peptides, and gray color indicates peptides with no difference. **(a)** BF/KF; **(b)** BX/KX; **(c)** BD/KD.

The differential peptides in all groups were evaluated using volcano plots. The BF-KF group showed significant upregulation (*p* < 0.05) of 272 peptides and downregulation of 582 peptides, with 854 peptides having no difference (Figure 5a). The BX-KX group showed significant upregulation of 952 peptides and downregulation of 701 peptides, with 1,653 having no difference (Figure 5b). The BD-KD group showed significant upregulation of 790 peptides and downregulation of 904 peptides, with 1,694 having no difference (Figure 5c). The peptides on the upper right and upper left sides showed substantial variations between the comparison groups.

### 5.1 Principal component analysis

**Figure 6:**
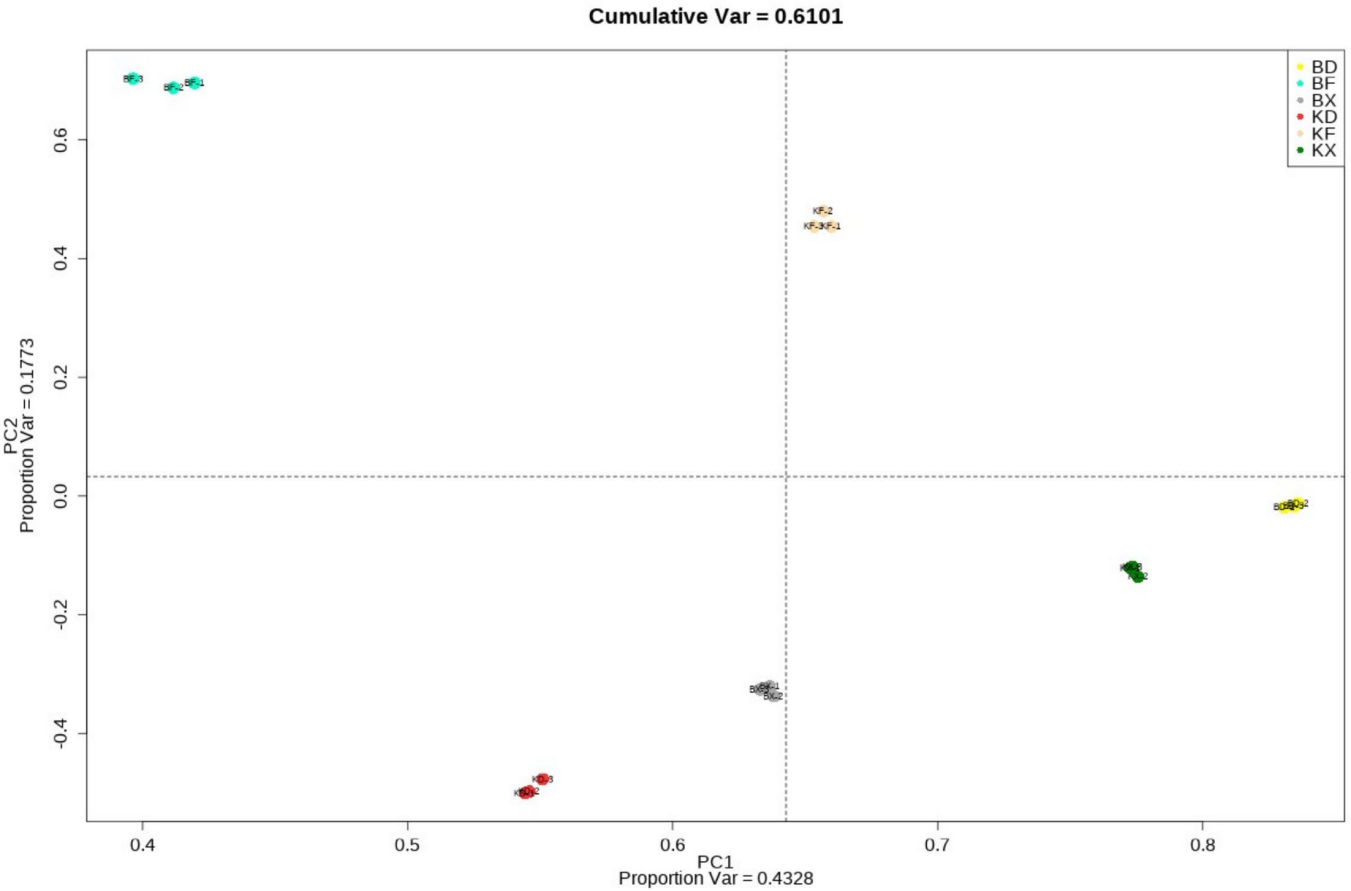
PCA analysis plot.

PCA is one of the most popular dimensionality reduction methods. Using orthogonal transformation, the quantitative information of a large number of peptides was transformed into group variables to draw PCA plots, which can visualize sample differences in spatial distribution. The smaller the difference in spatial distribution, the closer the data are, and each point in the PCA plot represents an experimental sample, and different colors were used to distinguish different groups. The PCA analysis plot in Figure 6 can be used to visualize the similarity of overall peptide quantification across samples, to test for overall quantitative differences between different experimental groups, and to evaluate and screen samples for quantitative anomalies within groups.

### 5.2 Cluster analysis: (heat map)

**Figure 7:**
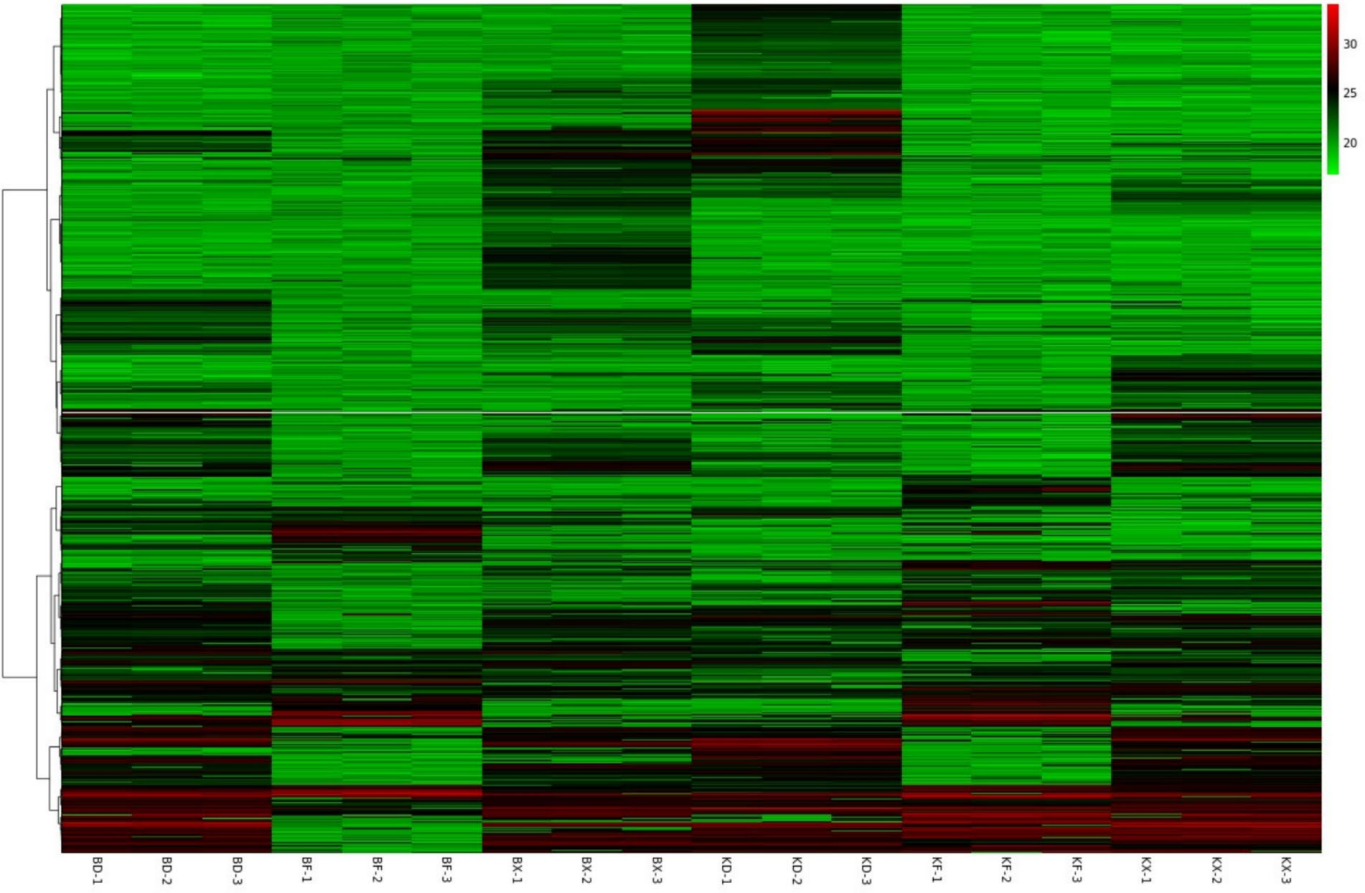
Heat map of peptides, color intensity indicating quantitative analysis of peptides in all groups.

HCA (Hierarchical clustering Analysis) was used for proteomics data mining to categorize peptides based on peptide expression profiles in individual samples and to observe the relationship between peptides, such as level of expression, pattern, repetition, etc. The results of all control and virus-induced groups’ differential peptides screened are shown in Figure 7. Each column was a sample, and each row was a peptide, where the color and its intensity indicate the quantitative data for that peptide. Peptides with close quantitative data patterns were located in similar rows.

### 5.3 GO annotation and enrichment analysis

**Figure 8:**
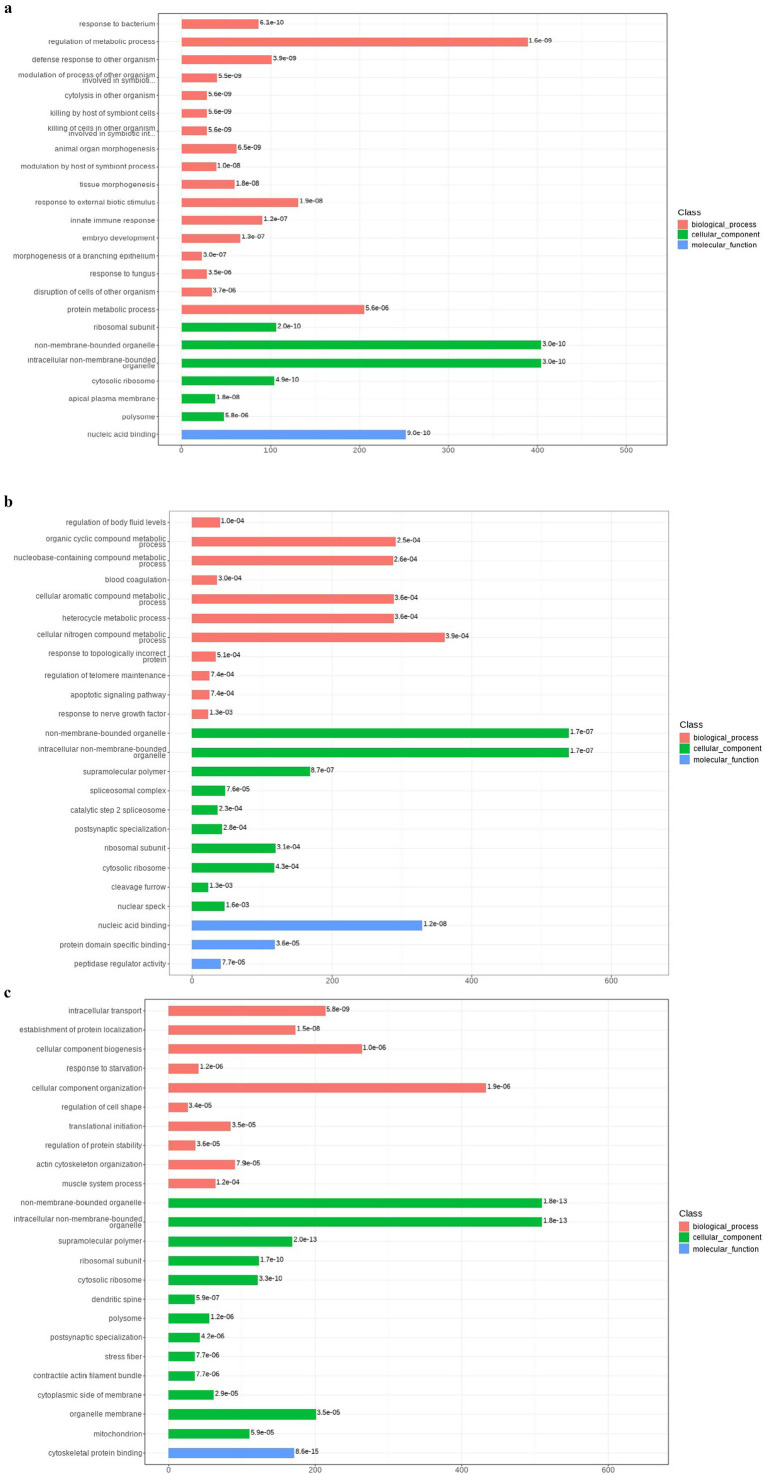
**(A)** GO annotation analysis of BF-KF group. **(B)** GO annotation analysis of BX-KX group. **(C)** GO annotation results of BD-KD group.

The Gene Ontology (GO) enrichment analysis was performed to figure out the protein enrichment. Enrichment was categorized by *p*-value (*p* ≤ 0.05), with smaller *p*-values indicating greater significance. Figure 8a represents the GO enrichment analysis of the BF-KF group and shows that the segments of differential proteins obtained by mass spectrometry were enriched in biological processes, cellular components, and molecular functions. GO annotation enrichment analysis revealed that proteins involved in biological processes, e.g., regulation of metabolic processes, proteins of cellular components like non-membrane bounded organelles, and proteins involved in molecular function, e.g., nucleic acid binding, were upregulated. Similarly, Figures 8b,c, represent the GO enrichment analysis of the BX-KX and BD-KD groups, respectively. So these up-regulated and down-regulated may have antiviral ability by the influence of immune response or other pathways, which was further assessed by employing bioinformatics tools like machine learning and deep learning.

### 5.4 KEGG annotation and enrichment analysis

**Figure 9:**
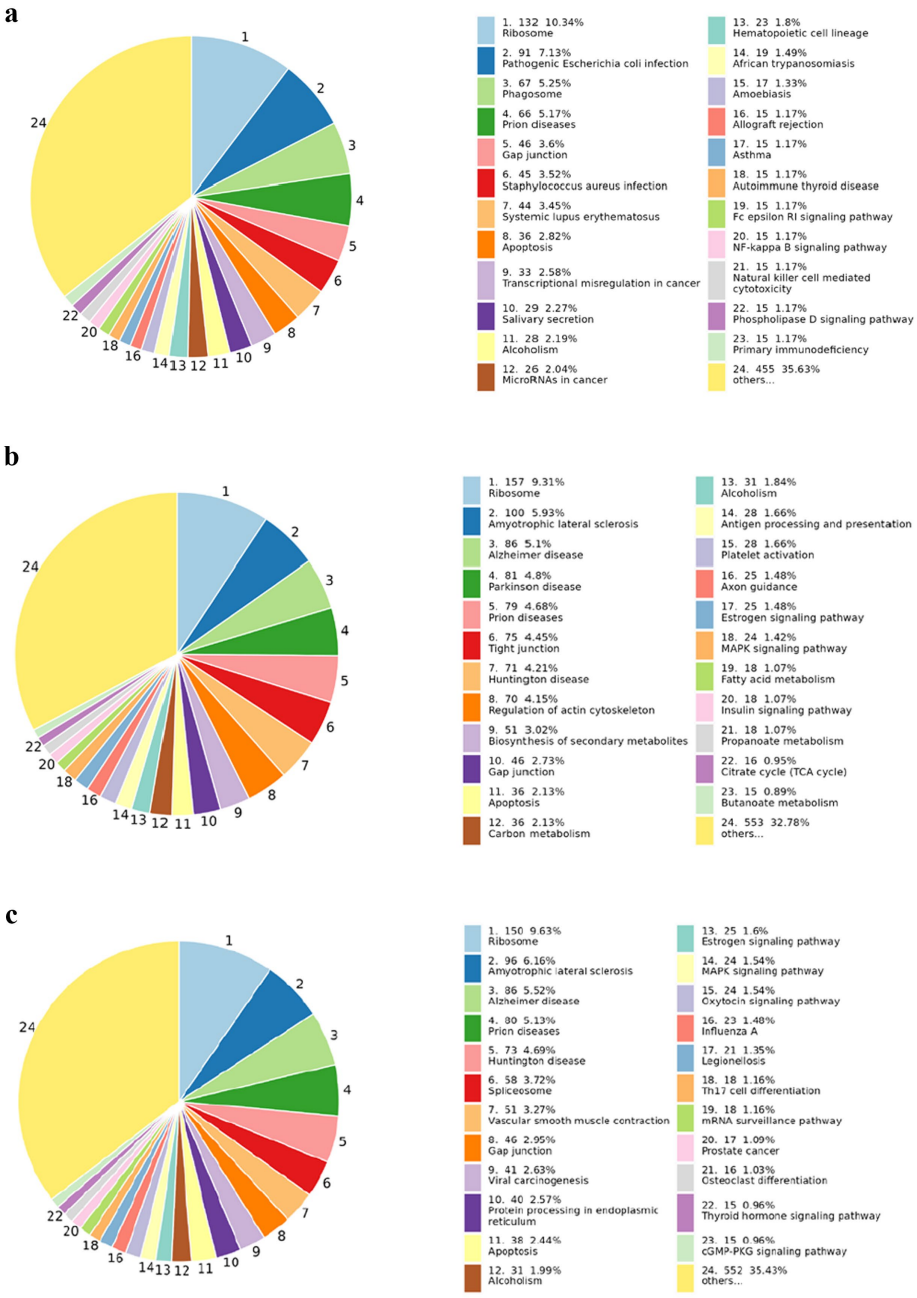
COG Enrichment analysis of all groups representing the enrichment of peptides involved in several cellular and signaling processes and other necessary processes. **(a)** Pie chart of BF-KF group; **(b)** Pie chart of BD-KD; **(c)** Pie chart of BX-KX Group.

KEGG (Kyoto Encyclopedia of Genes and Genomes) aids in the prediction of effective pathways involved in significant cellular biochemical processes. In this experiment, we analyzed KEGG annotations, comparison, and analysis of all the differential peptides in each of the comparison groups. Figure 9a shows the KEGG analysis of all the differential peptides identified in BF-KF. Differentially identified proteins were significantly enriched in phagosomes and apoptosis during cellular processes, and ribosomes in genetic information processing. Similarly, Figures 9b,c represent the outcomes of group BD-KD and BX-KX, respectively. So the suspected pathways of the antiviral peptides, which were predicted in this study by deep learning and machine learning, can be analyzed with the help of KEGG analysis.

### 5.5 COG annotation and PPI analysis

**Figure 10:**
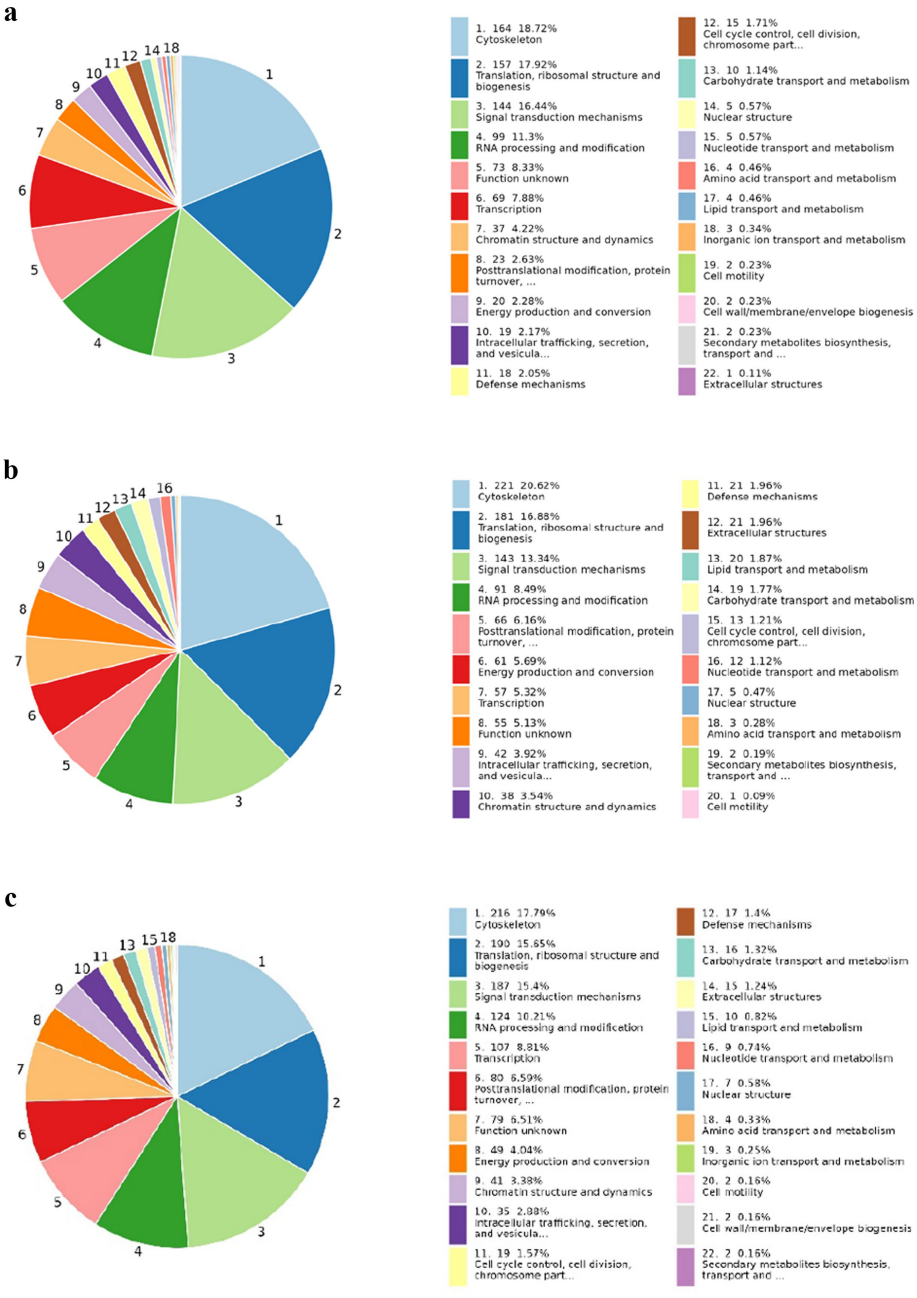
**(a)** Pie chart of BF-KF group **(b)** Pie chart of BD-KD group **(c)** Pie chart of BX-KX Group.

COGs (Cluster of Orthologous Groups)of proteins are constructed based on phylogenetic relationships of coding proteins in the complete genome. The comparison allows annotation of a protein sequence to a particular COG classification, and each COG cluster consists of an immediate homologous sequence, thus allowing the function of the sequence to be inferred. In this study, COG was annotated for different proteins in each separate group from which we collected samples. In Figure 10, from COG analysis of the groups, we found that the protein and peptide sequences involved in cytoskeleton formation, translation ribosomal structure and biogenesis, and signal transduction mechanism were significantly enriched, but the enrichment analysis percentage of the BD-KD group was the highest among all three groups. The protein–protein interaction analysis revealed the interaction of proteins, so that the interaction of predicted antiviral peptides with the other proteins and peptides can be assessed. The findings of the interaction analysis are shown in Supplementary Figure S1.

### 5.6 Antiviral peptides prediction

#### 5.6.1 Machine learning and deep learning model evaluation methods and results

Five-fold cross-validation is a typical implementation of k-fold cross-validation, and its core idea is to evaluate the generalization performance of the model more reliably through data partitioning and multiple iterations of the training-validation process. Specifically, this method randomly and evenly divides the original dataset into five subsets (or folds) of similar size that do not overlap. In each iteration, one of these subsets is selected as the validation set, while the remaining four subsets are used as the training set. Finally, the model performance is assessed based on the aggregated results from the five validation rounds. Compared to a single partition assessment method, five-fold cross-validation provides a more comprehensive reflection of the model’s stability under different data distributions, reducing evaluation variability caused by partition bias. In this study, we evaluated the accuracy, precision, recall, and F1 scores of the RF, SVM, and GNN models based on the mean results from five-fold cross-validation (48, 49). Additionally, to further assess the overall discriminative ability of the models, we visually compared the overall accuracy of the classifiers using receiver operating characteristic (ROC) curves and area under the curve (AUC) values, thereby validating the strengths and weaknesses of the different models from multiple perspectives. The formulas for the relevant evaluation metrics are as follows (Equations 1–4):


$$Recall=R=\frac{\mathrm{TP}}{TP+FN}\;$$ (1)

$$Precision=P=\frac{\mathrm{TP}}{TP+FP}$$ (2)

$$F1\_score=\frac{2\times P\times R}{P+R}\;$$ (3)

$$Accuracy=\frac{\mathrm{TP}+\mathrm{TN}}{TP+FP+FN+TN}\;$$ (4)

From the predicted outcomes presented in the table regarding accuracy, the random forest (RF) model demonstrated a relatively high accuracy of 0.955 on the training set, but its accuracy decreased to 0.877 on the validation set. The graph neural network (GNN) performed best on the training set; however, its validation metrics indicated that its generalization ability was not as robust as that of the RF. The support vector machine (SVM) achieved an accuracy of 0.925 on the training set and 0.866 on the validation set, revealing a performance gap compared to RF and GNN. In terms of precision, RF exhibited the highest values on both the training and validation sets, with scores of 0.993 and 0.928, respectively, signifying a strong capacity for correctly classifying positive samples. In contrast, SVM displayed lower precision, with values of 0.956 and 0.890 for the training and validation sets, respectively. Regarding recall, RF attained a recall of 0.914 on the training set while its recall on the validation set was lower at 0.810, whereas GNN showcased competitive recall rates, achieving 0.956 and 0.872 for the training and validation sets, respectively. Finally, in terms of F1 scores, GNN excelled with scores of 0.962 on the training set and 0.870 on the validation set, indicating the best overall performance, followed by the RF model, while the SVM model showed relatively weaker results. Table 3 provides the training and validation analysis values for the RF, SVM, and GNN models.

**Table 3:** Training, validation accuracy, and precision of RF, SVM, and GNN models.

Model	Accuracy		Precision		Recall		F1	
	Train	Valid	Train	Valid	Train	Valid	Train	Valid
RF	0.955	0.877	0.993	0.928	0.914	0.810	0.952	0.865
SVM	0.925	0.866	0.956	0.890	0.887	0.829	0.920	0.857
GNN	0.962	0.870	0.969	0.871	0.956	0.872	0.962	0.870

#### 5.6.2 Amino acids feature importance

**Figure 11:**
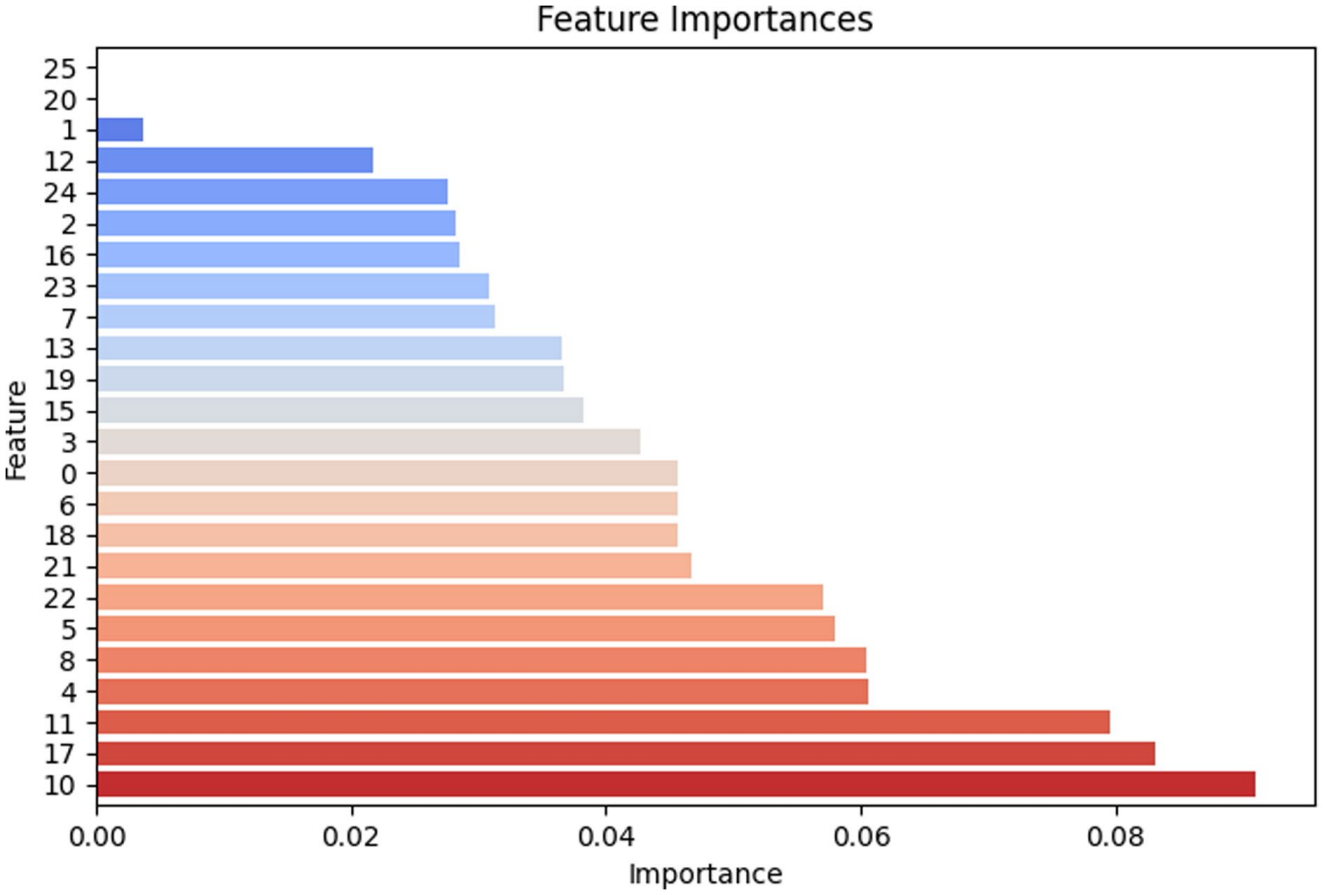
Feature importance chart of essential amino acids involved in the composition of peptides.

Figure 11 elaborates on the decision weights (feature importance ranking) of various amino acid categories in the random forest model when identifying viral peptide/antiviral peptide types based on amino acid combinations. This reflects the frequency and occurrence of amino acid repetitions in the formation process of relatively sensitive antiviral peptides.

From the feature importance ranking chart, it is clear that feature 10 (K), feature 17 (R), and feature 11 (L) have the highest importance, each close to 0.1, indicating that they play a crucial role in model predictions. Feature 4 (E), feature 8 (I), feature 5 (F), and feature 22 (W) also have relatively high importance, around 0.06. In contrast, feature 1 (A), feature 20 (U), and feature 25 (Z) have lower importance, below 0.01, suggesting that their contribution to the model was limited.

#### 5.6.3 Correlation heatmap

**Figure 12:**
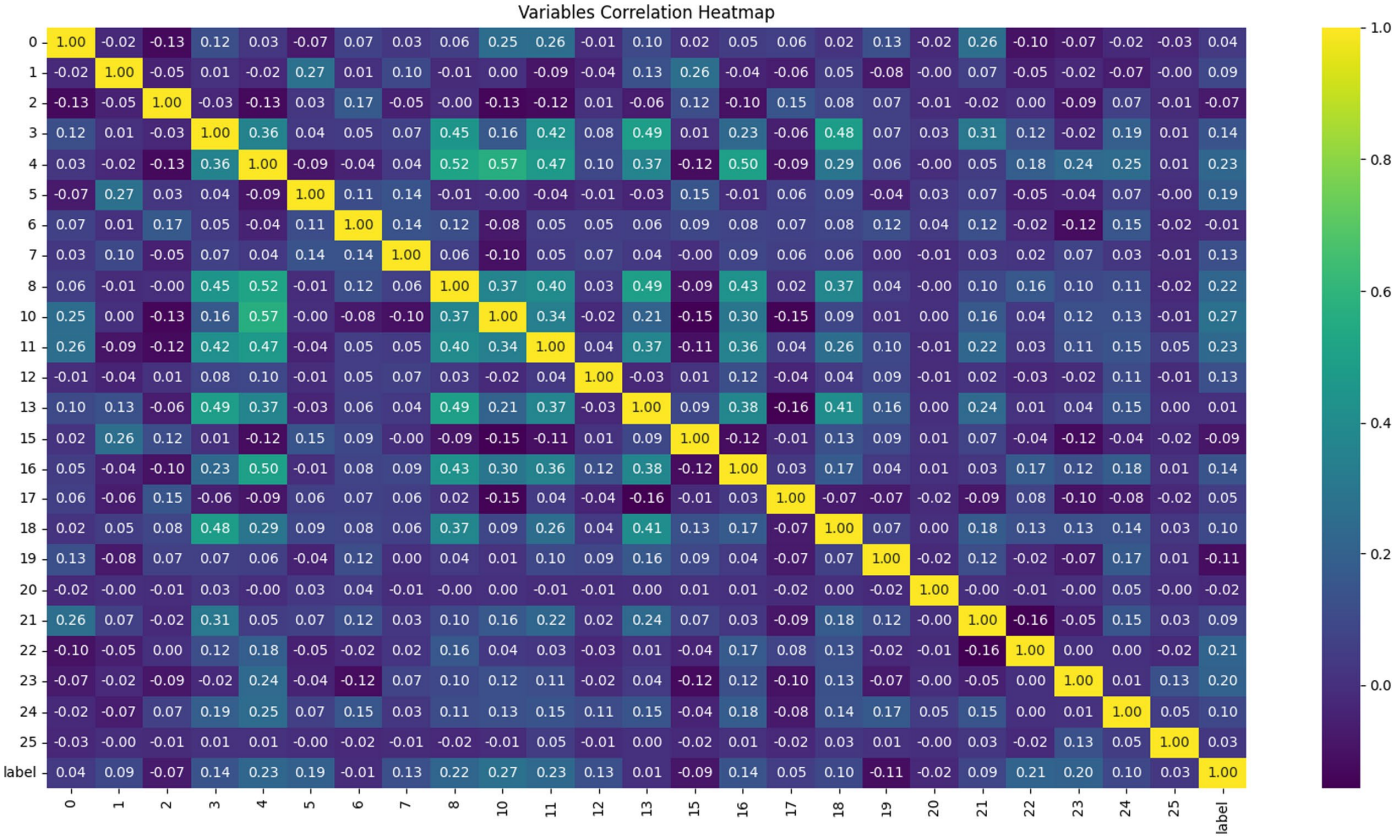
Correlation heat map elaborating the highest relationship of amino acids regarding the amino acids composition feature of antiviral peptides in the machine learning and deep learning models training set.

The correlation heatmap in Figure 12 demonstrates that the positive correlation between feature 4 (E) and feature 10 (K) is the most pronounced, reaching 0.57. This is followed by the positive correlation between feature 4 (E) and feature 8 (I), which is 0.52. The third is the positive correlation between feature 4 (E) and feature 16 (Q), which is 0.50. Notably, the negative correlations among all features are not significant, with the most extreme negative correlations being only −0.16 (between feature 13 and feature 17) and −0.15 (between feature 10 and both feature 17 and feature 15). It is important to note that feature 4 (E) shows the closest associations with other features, possibly indicating it is a foundational amino acid segment. Finally, the most prominent positive correlation with the label is seen in feature 10 (K), while the most significant negative correlation is in feature 19 (T), with values of 0.27 and −0.11, respectively. This aligns to some extent with the conclusions drawn from the feature importance ranking.

#### 5.6.4 ROC curve comparison

**Figure 13:**
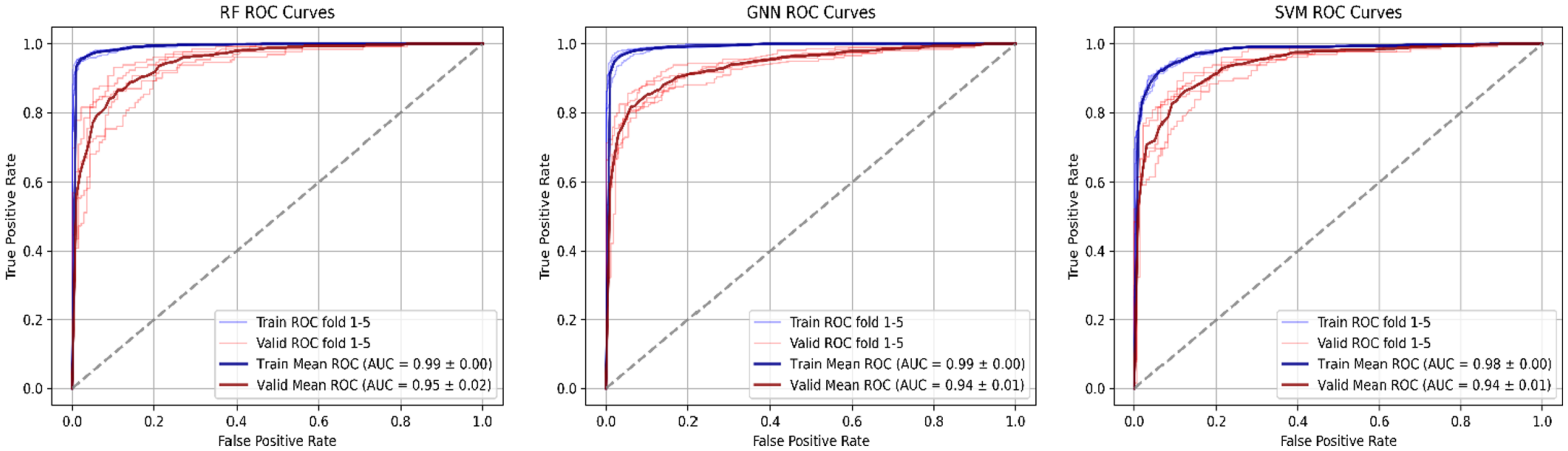
Graphical view of training ROC and validation ROC accuracy of RF, GNN, and SVM models.

Ultimately, as shown in Figure 13, through comparative analysis of the ROC curves, it is evident that the RF model demonstrates the best performance, both in training data and in prediction. The AUC for the RF model in training was 0.99, and the predicted AUC was 0.95 ± 0.02. The GNN model performed comparably, with a training AUC of 0.99 and a prediction AUC of 0.94 ± 0.01, indicating it does not generalize as well as the RF model. The SVM model exhibited a training ROC area of 0.98 and a prediction ROC area of 0.94 ± 0.01, which is satisfactory but inferior to both the RF and GNN models. Therefore, considering the evaluation results mentioned above, the RF model is selected to predict the labels of the prediction set.

### 5.7 Comparative analysis

The comparative evaluation of the computational models developed in this study for antiviral peptide (AVP) prediction highlights their strong predictive power and consistency. Among all the models, the Random Forest (RF) exhibited the best performance, achieving a validation accuracy of 0.877 and a validation precision of 0.928, demonstrating excellent feature discrimination and robustness. The Support Vector Machine (SVM) also produced competitive results, with a validation accuracy of 0.866 and precision of 0.890, confirming its effectiveness in classifying complex peptide patterns. Likewise, the Graph Neural Network (GNN) attained a validation accuracy of 0.870 and precision of 0.871, emphasizing its ability to capture both sequential and structural relationships within peptide data.

**Figure 14:**
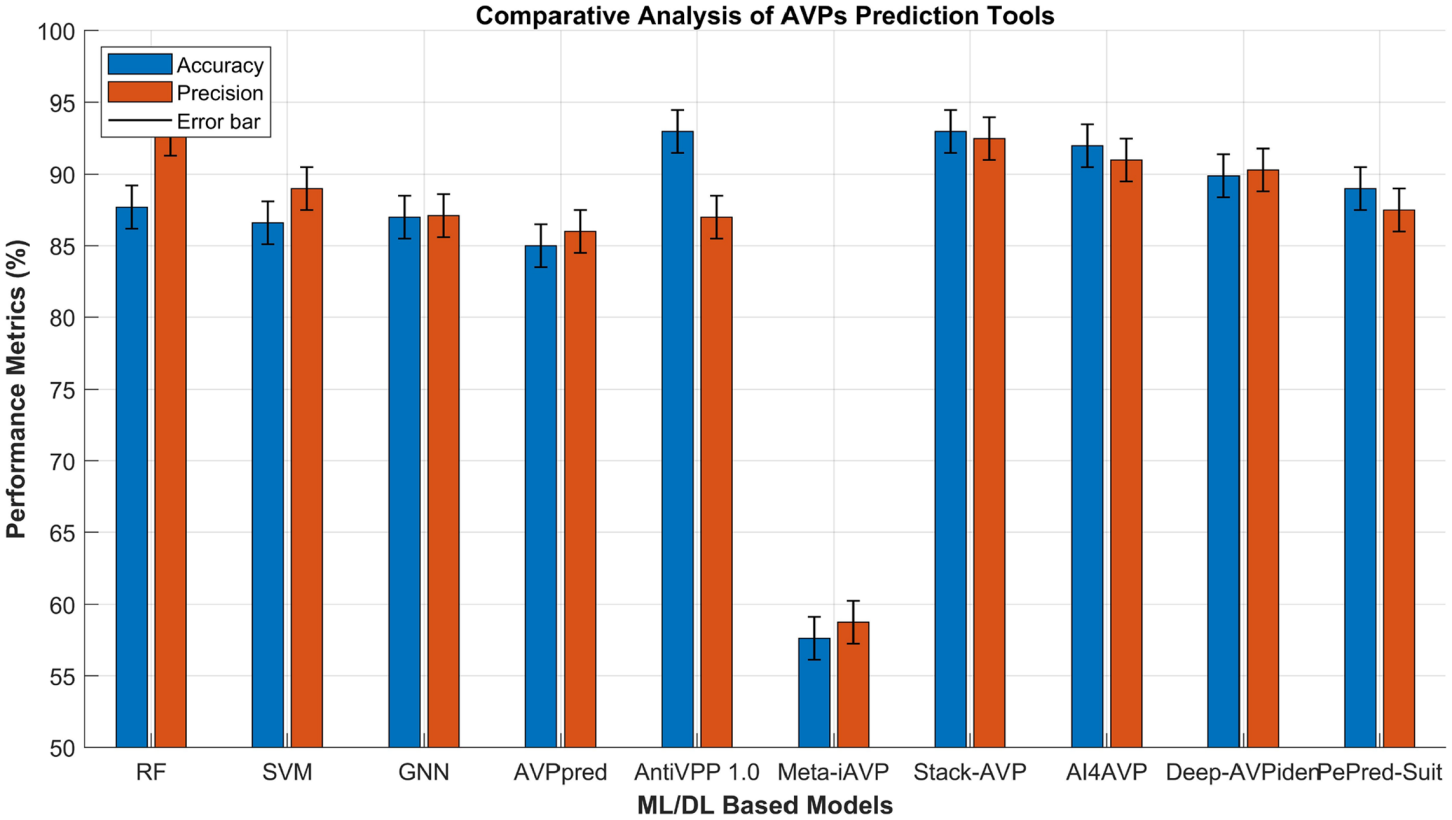
Comparative performance analysis of different machine learning and deep learning models for antiviral peptide (AVP) prediction. The figure illustrates the validation accuracy and validation precision of the models developed in this study, Random Forest (RF), Support Vector Machine (SVM), and Graph Neural Network (GNN), compared with other existing AVP prediction tools.

In comparison with previously reported tools (28) (Figure 14), the proposed models achieved results that are on par with, or in some cases superior to, leading predictors such as Stack-AVP and AntiVPP1.0, despite employing simpler and more interpretable architectures. Importantly, both RF and GNN models demonstrated consistent performance without the need for extensive hyperparameter optimization or ensemble strategies, suggesting that well-tuned traditional and graph-based methods can deliver high accuracy with lower computational demands. Moreover, the balanced outcomes across accuracy and precision indicate strong generalization and a reduced tendency toward overfitting. Overall, these findings highlight the reliability, scalability, and interpretability of the proposed models and support the conclusion that thoughtfully designed machine learning and graph-based frameworks can provide efficient, transparent, and high-performing alternatives to complex ensemble approaches for antiviral peptide prediction.

## 6 Discussion

Mass spectrometry-based proteomics has significantly enhanced the comprehension of the intricate molecular foundations of human health and disease (50). Contemporary experimental methods predominantly integrate automated methodologies for protein identification, characterization, and quantification, thereby enhancing the portability and reliability of MS-based proteomic analysis upstream. Recent improvements in multiplexing technology facilitate the examination of several samples with enhanced throughput (10, 51). Proteomics analysis provides us with an insightful study of the dysregulated pathways followed by several peptides in numerous viral diseases, like Porcine respiratory syndrome virus. PRRSV inhibits IFN-*β*/RIG-I–MAVS signaling and manipulates TBK1 and downstream IFN responses (11). Simultaneously, antiviral peptides or other therapeutic proteins also follow several mechanistic immunogenic, autophagy (52), mitochondrial, lysosomal, or other cellular pathways. Antiviral peptides regulate interferon responses and engage with interferon-stimulated effectors (ISGs); ISG15 is a well-documented antiviral effector that associates ubiquitin-like alteration with innate antiviral action. Consequently, these pathways serve as standard indicators of antiviral activity. Among these ISGs, the ubiquitin-like protein ISG15 is one of the most significantly and swiftly produced, with recent studies demonstrating its capacity to directly limit viral propagation and influence host immunity (53) Similarly, Numerous antimicrobial peptides (AMPs), such as LL-37, have been demonstrated to activate or alter autophagy in infected host cells; autophagy serves as a prevalent antiviral effector mechanism and is targeted by both viruses and host defense peptides (54). AVPs/AMPs can influence apoptotic signaling (either promoting apoptosis of infected cells or inhibiting excessive cell death) as part of their immunomodulatory role against viral diseases (55, 56).

Considering the emergence of antiviral drug resistance, it is imperative to identify novel and effective antiviral agents. The potency, effectiveness, and pharmacokinetics render antiviral peptides (AVPs) attractive therapeutic agents. Computational methods are essential for the rapid and precise identification of AVPs in the post-genomic era, owing to the increasing volume of peptide sequences. The identification of peptide-based medications necessitates the utilization of efficient machine learning algorithms such as Random Forest (RF) and Support Vector Machine (SVM) (57). Innovative techniques that enhance ion sources, spectral resolution, and dynamic detectors with a broader spectrum could potentially influence the progression of upstream proteomics (51). This study utilized mass spectrometry (MS) to identify differentially expressed peptides, which were then used as a dataset for training machine learning (ML) and deep learning (DL) models to predict potential antiviral peptides (AVPs). This technique facilitated the integration of high-throughput peptide profiling with sophisticated computational methods, yielding an effective tool for identifying antiviral candidates from intricate proteomic data. The application of machine learning and deep learning prediction models facilitated an in silico evaluation of their prospective antiviral characteristics. The connection between differentially identified peptides and computationally predicted AVPs indicates a significant relationship that underscores the biological importance of the predictions.

The necessity for antiviral therapies is critical, and small molecule-based antiviral peptides offer a potentially efficacious treatment alternative. The accuracy of AVP predictions is essential for the advancement of peptide-based therapeutics. Consequently, to enhance the effectiveness of the AVP’s prediction, we introduced a computational predictor, particularly a random forest model. Model training necessitates the utilization of optimal physicochemical characteristics, amino acid composition (AAC), and secondary structure, which are significant for the prediction of antiviral peptides (AVPs) (58). The essential role of AAC (amino acid composition) in antiviral peptides was evaluated using experiment-based data. Lysine is recognized as the most crucial residue in antiviral peptides and the predominant residue in differentiating AVPs. Our tests indicated that the AVPs were rich in leucine, lysine, and glutamic acid. This study was the inaugural application of GNN, SVM, and RF in (AVP’s) prediction connectedly. The deep learning GNN model has been used in earlier studies in the prediction of antimicrobial peptides (59). Being pioneers, we used the GNN model first ever in the prediction of antiviral peptides. Our predictive models were based on amino acid content, aliphaticity, hydrophilicity, fold change/log2 value, and secondary structure. AAC encapsulates the overarching chemical and compositional trends of peptides, facilitating a fundamental comprehension of residue frequency patterns typical of antiviral peptides and simultaneously strengthening the importance of their context within the whole protein. Nonetheless, amino acid composition in isolation fails to elucidate the organization or interactions of residues within the peptide’s structural framework. To rectify this, secondary structure features were integrated, which elucidate the local folding patterns and conformational propensities of the peptide sequences. Furthermore, hydrophilicity features impart knowledge regarding residue-specific physicochemical properties vital for peptide stability, membrane interaction, and prospective antiviral efficacy. The results indicated that the RF models successfully predicted AVPs with the highest accuracy based on fundamental physicochemical features. Our analysis of the independent test data supplied by *Thakur et al.*’s study demonstrated that RF outperformed SVM and GNN in differentiating AVPs based on these physicochemical parameters. Our findings indicate that RF, a formidable classifier, excels in numerous challenges, and GNN can also be used as an antiviral peptide predictor. The high accuracy level of all three designed models demonstrates the ideality of the models in PRRSV (AVP’s) prediction, which can be a baseline and source of novel therapeutics discovery.

In AVP’s prediction, to pave new scientific domains, integrating epitope-based knowledge can markedly improve the biological interpretability and therapeutic efficacy of computationally discovered sequences. Knowing cell epitopes is crucial for future research on the roles of structural proteins associated with PRRSV and for the advancement of novel diagnostic techniques. Epitopes can be classed into B-cell and T-cell epitopes depending on receptor cells, and further categorized as linear or conformational epitopes according to the spatial structure of the antigen. Future research may benefit from integrating known epitope-containing peptide sections to enhance the rational selection of membrane-permeable sequences. Recent investigations have delineated immunodominant and structurally conserved epitopes within PRRSV glycoproteins and nucleocapsid proteins, offering significant templates for the production of physiologically pertinent peptides (60). Comprehensive epitope atlases have delineated conserved peptide fragments within GP and N proteins, characterized by specific biophysical properties and structural accessibility (61), whereas immunoinformatics-based screening of GP3 and GP5 has underscored regions exhibiting elevated antigenicity and potential surface exposure (62). Epitope mapping of the N protein with monoclonal antibodies revealed the impact of particular sequence motifs on immunological recognition and protein stability. Incorporating epitope-derived information in future iterations of this type of work may facilitate the selection of membrane-compatible peptides that retain biologically significant epitopic characteristics.

## 7 Conclusion

Proteomics analysis revealed a wide range of peptides database influenced by PRRSV, mostly associated with cellular functions. Bioinformatics tools, including ML and DL, were employed to identify composition-based antiviral peptides. This research employed a deep learning GNN model for the first time to predict antiviral peptides. RF model demonstrated the highest 0.95 ± 0.02% accuracy and reliability than GNN and SVM models. PRRSV antiviral peptide discovery and prediction have been scarce. This study highlights the significance of lysine and *α*-helical secondary structures in antiviral peptides. Therefore, this study will be a foundational element in the exploration of therapeutics targeting PRRSV. To promote the progress of antiviral peptides in the pharmaceutical field, creating a database was crucial. In this study, a comprehensive library of AVPs was established to function as a significant resource for the discovery, design, and experimental validation of novel antiviral peptides targeting PRRSV.

## Data Availability

The datasets of the PRRSV antiviral peptides of this study are available at https://github.com/Wafa-Yousaf/PRRSV-AVPeP-ML-Omics.git.
