# Supplementary material for: Data-driven discovery of antiviral peptides against PRRSV using multiple machine learning models
Source: Front Vet Sci. 2025 Dec 5;12:1681083. doi: 10.3389/fvets.2025.1681083 (PMC12714607; doi:10.3389/fvets.2025.1681083)
Supplement: Supplementary file 1 [file Table_1.DOCX]

### S1: Interaction network analysis

Proteins normally don’t work alone, the exercise of their actions must depend on the regulation and mediation of other proteins. This regulation and mediation requires the existence of direct and indirect interaction between proteins. The protein interaction network of all the differential peptides detected was predicted based on the STRING database. The protein-protein interaction results of differential proteins of BF-KF, BD-KD, and BX-KX groups are shown in figure 8 in which each node is a screened differential protein, and the connecting lines between the nodes indicate the existence of known or predicted possible protein interaction between the two proteins. The node color indicates the quantitative information of the protein in the comparator group. Red indicates upregulation, blue indicates downregulation, and the purple color indicates that the protein is partially upregulated and partially downregulated.


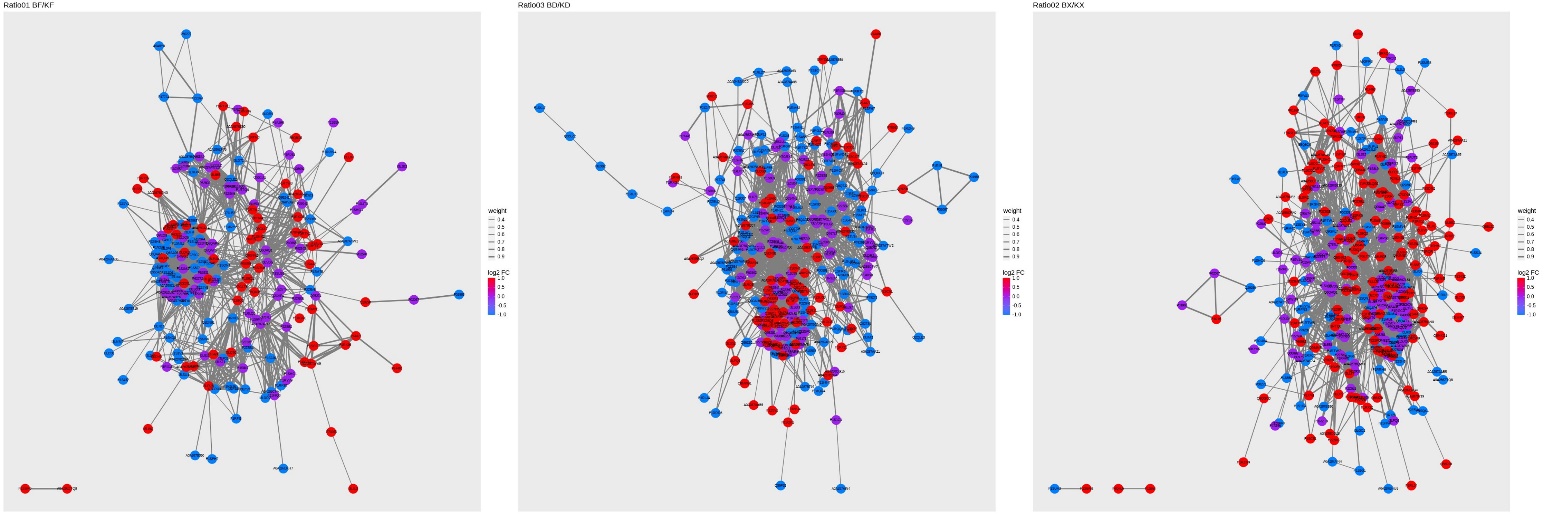


BF-KF BD-KD BX-KX

**Figure S1**: Protein-protein interaction network analysis based on String Database
