# Supplementary material for: Data-driven discovery of antiviral peptides against PRRSV using multiple machine learning models
Source: Front Vet Sci. 2025 Dec 5;12:1681083. doi: 10.3389/fvets.2025.1681083 (PMC12714607; doi:10.3389/fvets.2025.1681083)
Supplement: Supplementary file 2 [file Table_2.DOCX]

**Supplementary file S2**

**Group 1** (Participation in the formation of specified amino acids predominantly involved in antiviral peptides)**

**K,L,T,I,R,N,S,W,V,A,P,E,D,G,C,Y,Q,F,H,M.**

**Group 2** Participation in hydrophilic interactions,

**R,K,E,Q,D,N,H,P,Y,S,T,G.**

The hydrophobicity of the antiviral peptide is primarily attributed to the facilitative interactions with lipid membranes and enhances peptide–membrane affinity.

**Group 3** (Participation in the formation of secondary structure, directly acting on viral proteins)**

**L, A, K, M, E.**

Peptides with an abundance of the above-mentioned amino acids adopt a well-defined secondary structure, which contributes to its stability and effective binding to target membranes or viral components.

**Group 4** (Participation in polar interactions such as hydrogen bonding, stabilizing antiviral conformations)**

**- Glutamine (Q)**

**- Asparagine (N)**

**- Serine (S)**

**- Threonine (T)**

**- Glycine (G)**

**- Selenocysteine (U)**

These polar amino acids can stabilize the spatial conformation of antiviral peptides through interactions such as hydrogen bonding, enabling better binding to viral targets. Selenocysteine, as a special amino acid, may play a role in some peptides with antioxidant and antiviral activity.
